# Dataset for human fall recognition in an uncontrolled environment

**DOI:** 10.1016/j.dib.2022.108610

**Published:** 2022-09-17

**Authors:** José Camilo Eraso Guerrero, Elena Muñoz España, Mariela Muñoz Añasco, Jesús Emilio Pinto Lopera

**Affiliations:** a Ingeniería Electrónica y Telecomunicaciones, Universidad del Cauca, Popayán, Colombia; b Ingeniería de Sistemas, Universidad de la Amazonia, Florencia, Colombia

**Keywords:** Fall detection, Activities of daily living, Feature extraction, YOLO, Openpose, Uncontrolled environment

## Abstract

This article presents a dataset (CAUCAFall) with ten subjects, which simulates five types of falls and five types of activities of daily living (ADLs). Specifically, the data include forward falls, backward falls, lateral falls left, lateral falls right, and falls arising from sitting. The participants performed the following ADLs: walking, hopping, picking up an object, sitting, and kneeling. The dataset considers individuals of different ages, weights, heights, and dominant legs. The data were acquired using an RGB camera in a home environment. This environment was intentionally realistic and included uncontrolled features, such as occlusions, lighting changes (natural, artificial, and night), participants different clothing, movement in the background, different textures on the floor and in the room, and a variety in fall angles and different distances from the camera to the fall. The dataset consists of 10 folders, one for each subject, and each folder includes ten subfolders with the performed activities. Each folder contains the video of the action and all the images of that action. CAUCAFall is the only database that contains details of the lighting lux of the scenarios, the distances from the human fall to the camera and the angles of the different falls with reference to the camera. The dataset is also the only one that contains labels for each image. Frames including human falls recorded were labeled as ``fall'', and ADL activities were marked ``nofall”. This dataset is useful for developing and evaluating modern fall recognition algorithms, such as those that apply feature extraction, convolutional neural networks with YOLOv3-v4 detectors, and camera location and resolution increase the performance of algorithms such as OPENPOSE. Thus, the dataset enables knowledge of the real progress of research in this area since existing datasets are used in strictly controlled environments. The authors intend to contribute a dataset with real-world housing environments characteristics.

**Specifications Table**

**Table:** 

Subject	Computer Science
Specific subject area	Human fall recognition by computer vision in uncontrolled environments mainly focuses on YOLOv3-v4 detectors [1,2].
Type of data	Video Image Text file (.txt)
How the data were acquired	The data were obtained with a single camera located in the upper corner of the stage, covering a large field of view to monitor the user's activity. The camera captured videos with changing lighting or without light. The data were stored in a DVR programmed to detect and record motion. The frame labels, which contain the information about the activities and segment each image between ``fall'' and ``nofall'', were manually created with a text editor.
Data format	Raw and analyzed
Description of data collection	The dataset was designed to recognize human falls in an uncontrolled home environment, with occlusions, changes in lighting (natural, artificial, and night), variety in participants’ clothing, movement in the background, different textures on the floor and in the room. The dataset is the only one that provides the lux of illumination of the scenarios, the distance from the human fall to the camera, and the angles of the different falls with reference to the camera, and provides participants of different ages, weights, heights, and even dominant legs. This dataset contributes to the real progress of research in recognizing falls. In addition, the proposed dataset is the only one that contains segmentation labels for each of its images. These labels serve to implement human fall recognition methods employing YOLO detectors.
Data source location	• Institution: Universidad del Cauca • City/Town/Region: Popayán, Cauca • Country: Colombia • Latitude and longitude for collected samples/data: 2° 25′ 59″N 76° 37′ 1″O.
Data accessibility	The datasets are publicly and freely available on mendeley data repository with doi:10.17632/7w7fccy7ky.4 at https://data.mendeley.com/datasets/7w7fccy7ky/4[3]

## Value of the Data

**Fig. 1:**
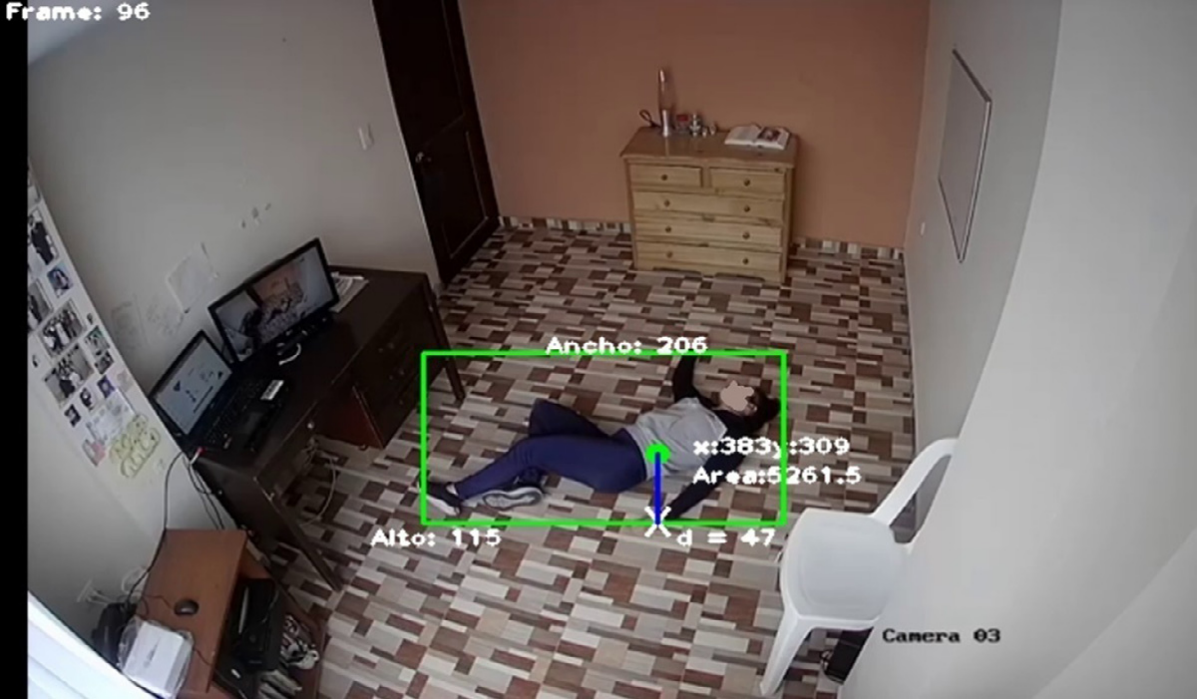
Fall recognition based on Feature Extraction.

**Fig. 2:**
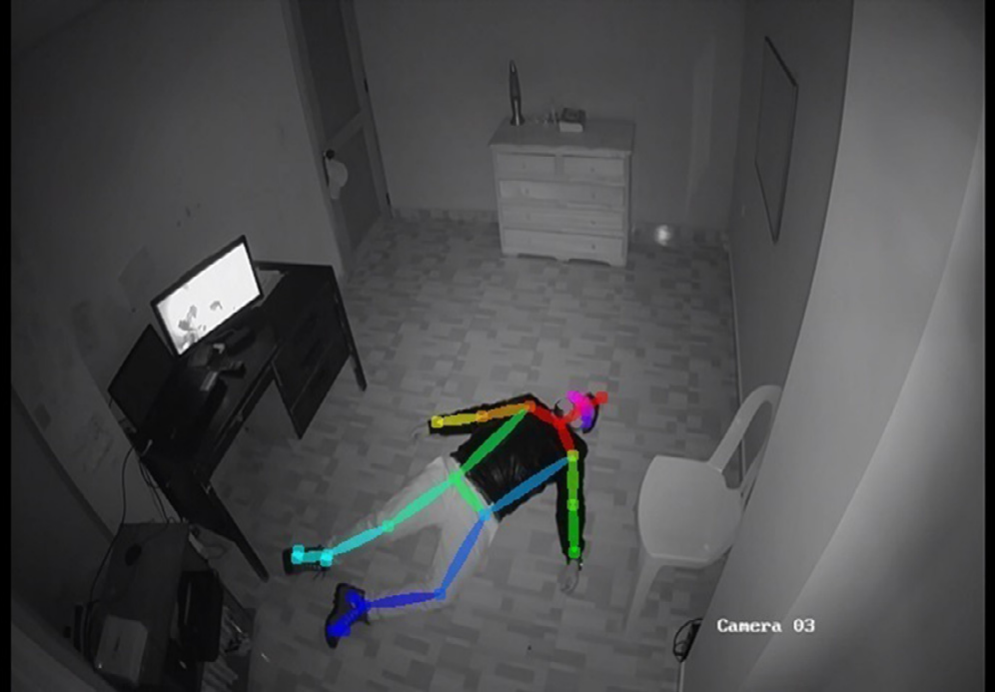
Fall recognition based on OPENPOSE.

**Fig. 3:**
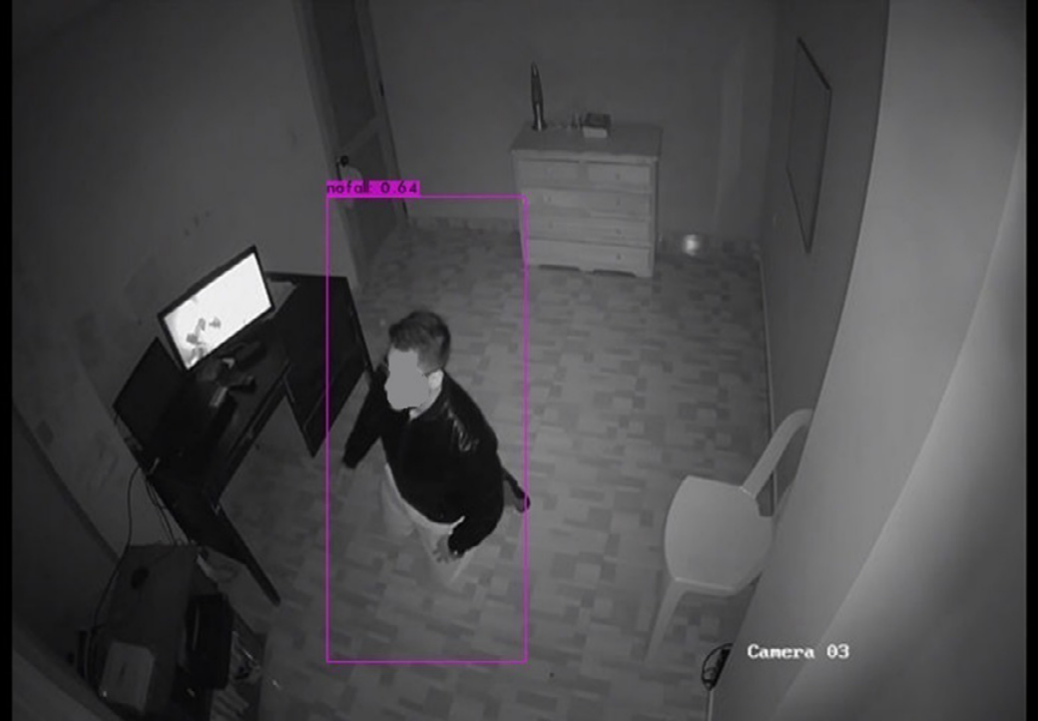
Fall recognition based on YOLO detectors.

• Applications of fall recognition by computer vision have obtained satisfactory results. However, the datasets used have restricted environments and the falls are simulated, which is controversial. Algorithms trained with highly controlled databases do not perform well in predicting real falls [4,5]. For this reason, CAUCAFall is proposed, this dataset can be utilized to analyze the real progress of human fall recognition by evaluating the behavior of fall recognition algorithms in an uncontrolled environment that simulates a realistic environment. Furthermore, the dataset provides labels for each image, useful for training and operating YOLO detectors, in addition, the resolution and angle at which CAUCAFall was recorded allow high performance in modern algorithms that detect the human bone map and can be used for human fall recognition, such as OpenPose. These are new artificial vision methods applicable to the recognition of human falls.
• The dataset is in the public domain, benefiting the entire scientific community that wishes to evaluate its fall recognition algorithms in uncontrolled environments to create robust algorithms that benefit society, especially the elderly. Worldwide, falls are the second most common cause of accidental deaths and one of the leading causes of injury or disability. In the United States, every 11 s, an elderly person who has fallen is taken to an emergency room, and every 19 min, one of these people dies. As the elderly population grows, this rate will rise, and by 2030, seven adults are predicted to die from a fall in the United States [6,7].
• The dataset can be used for training, validation, and testing of any human fall recognition method using computer vision to evaluate the method's performance in uncontrolled environments. Furthermore, the dataset mainly uses the images and videos to train convolutional neural networks or methods involving feature extraction (see Fig. 1). In addition, the dataset is useful for evaluation in uncontrolled environments of novel fall recognition methods, such as those based on OPENPOSE [8] that extract the bone map of the human silhouette from 2D images (see Fig. 2). Furthermore, one can combine the visual data with the image labels to implement algorithms that work with YOLO detectors (see Fig. 3).

## 1 Data Description

The proposed dataset is the only one that contains segmentation labels for each of its images, which serve to implement human fall recognition methods by YOLO detectors. Moreover, compared to other datasets [9], [10], [11], [12], [13], [14], [15], [16], this dataset was developed with a single camera in a home environment. This environment was intentionally realistic and included uncontrolled features. Table 1 compares the most popular databases among the scientific community.

**Table 1:** comparison of datasets for human fall recognition.

Dataset	Year	Camera	Light condition	Occlusion	Variety in fall angles	Different distances	File formats	Labels for YOLO	OpenPose performance	Lux	Angle details	Distance details	Availability (June,2022)
Multiple cameras fall dataset [9]	2010	RGB	artificial	**✓**	–	–	.avi	–	**86.5%**	–	–	–	**✓**
Le2i [10]	2012	RGB	natural, artificial	**✓**	**✓**	–	.avi	–	–	–	–	–	–
SDUFall [11]	2014	Kinect	natural, artificial	–	–	–	Depth videos .avi	–	–	–	–	–	–
EDF&OCCU [12]	2014	Kinect	artificial	**✓**	–	**✓**	.txt	–	–	–	–	–	–
UR Fall Detection [13]	2014	Kinect	artificial	–	–	**✓**	avi. csv	–	**87.93%**	–	–	–	**✓**
FUKinect-Fall [14]	2016	Kinect	–	–	**✓**	**✓**	Depth videos. csv	–	**85.4%**	–	–	–	**✓**
Fall Detection Dataset [15]	2017	RGB Kinect	natural, artificial	–	**✓**	–	png. csv	–	**92.72%**	–	–	–	**✓**
UPFall [16]	2019	RGB	natural, artificial	–	**✓**	–	png. csv	–	**71.28%**	–	–	–	**✓**
CAUCAFall [3] (Ours)	2022	RGB	natural, artificial, no light	**✓**	**✓**	**✓**	Jpeg .txt. avi	**✓**	**97.84%**	**✓**	**✓**	**✓**	**✓**

CAUCAFall (see Table 1) using a single camera has the main characteristics of uncontrolled environments: changing light conditions, occlusions, varying fall angles, and falls at different distances from the camera. In addition, CAUCAFall is the only dataset containing fall and no-fall labels to be used in YOLO detectors as a novel detection and recognition method, is the only database that details camera distances to human fall and fall angles with reference to camera position, and also details the illumination lux of different environments. In addition, the authors found that CAUCAFall's camera location and resolution increase the performance of current human bone map detection algorithms, such as OpenPose, which can contribute to the advancement of human activity recognition in different environments.

Ten subjects (see Table 2) simulated five types of falls and five types of activities of daily living (ADLs). The data included forward falls, backward falls, lateral falls left, lateral falls right, and falls arising from sitting. The participants’ ADLs were walking, hopping, picking up an object, sitting, and kneeling. Frames that recorded human falls were labeled ``fall'', and ADL activities were labeled ``nofall''. Frames were labeled ``fall'' only when the human body is on the ground because of a fall. The labels can be used for fall recognition by YOLO detectors and by feature extraction, for example, by determining the speed with which the body falls or by analyzing the area of the human silhouette and its radial spectrum (these data are not part of CAUCAFall).

**Table 2:** Characteristics of the participants.

Subject	Gender	Age	Weight (Kg)	Height (Meters)	Health Conditions	Dominant Leg	Outfit
1	Female	27	56	1.65	Healthy	Right	Gray jacket, blue pants, black shoes, hair tied.
2	Male	34	70	1.73	Healthy	Left	Red jersey, blue pants, white shoes.
3	Female	31	58	1.60	Healthy	Left	Brown jacket, gray pants, blue shoes, loose hair.
4	Male	38	75	1.68	Healthy	Right	Black jacket, blue pants, gray shoes, cap.
5	Male	40	67	1.70	Healthy	Right	Black jacket, brown pants, black shoes.
6	Male	33	77	1.65	Healthy	Right	Black jacket, white pants, brown shoes.
7	Female	23	54	1.59	Healthy	Right	Gray jersey, black pants, blue shoes, hair tied.
8	Female	25	59	1.63	Healthy	Right	Blue jersey, gray pants, brown shoes, hair tied.
9	Male	37	79	1.74	Healthy	Left	Yellow jersey, brown pants, brown shoes.
10	Female	28	61	1.62	healthy	Right	Green shirt, purple pants, black shoes, loose hair.

**Fig. 4:**
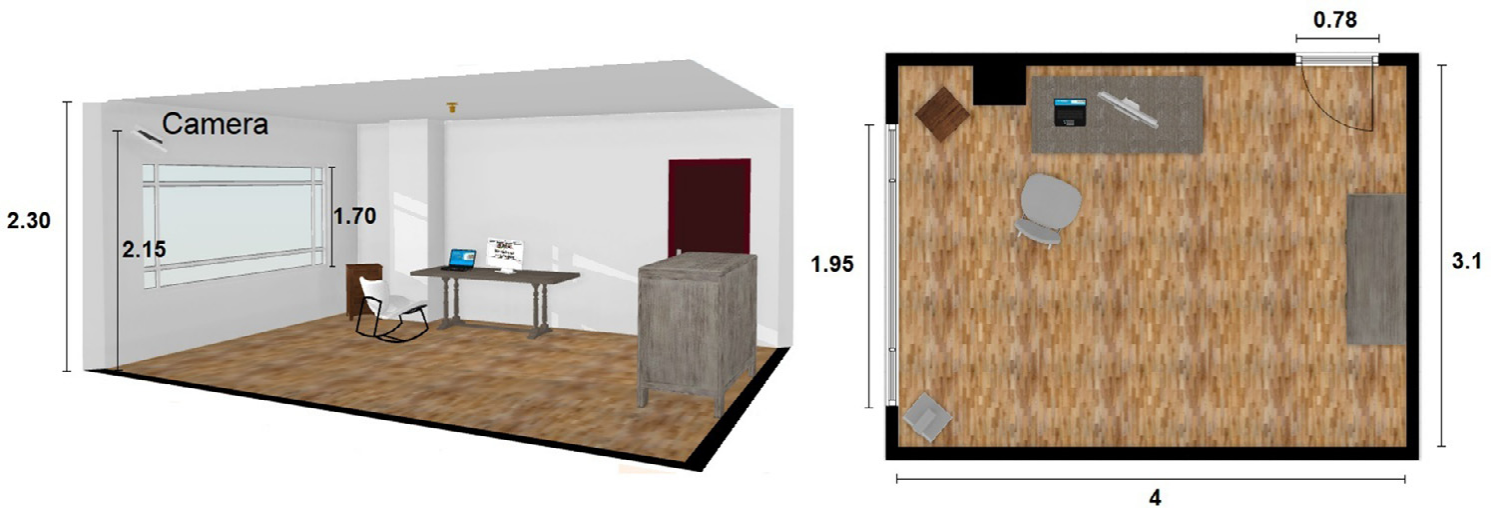
Scenario dimensions (in meters).

Fig. 4 provides a map with the dimensions of the scenario where human falls were simulated. In the scene, there is a window through which natural light enters. The recording camera is located at a height of 2.15 m.

In the fall simulation, the initial position of the participants is standing, with the exception of ``Fall sitting'' whose initial position is sitting.

The data are organized into 10 main directories corresponding to the subjects. Each directory contains 10 folders with the different performed activities. Each folder includes a video of the action in .avi format, images of the action in .png format, and each frame segmentation tags in .txt format.

**Fig. 5:**
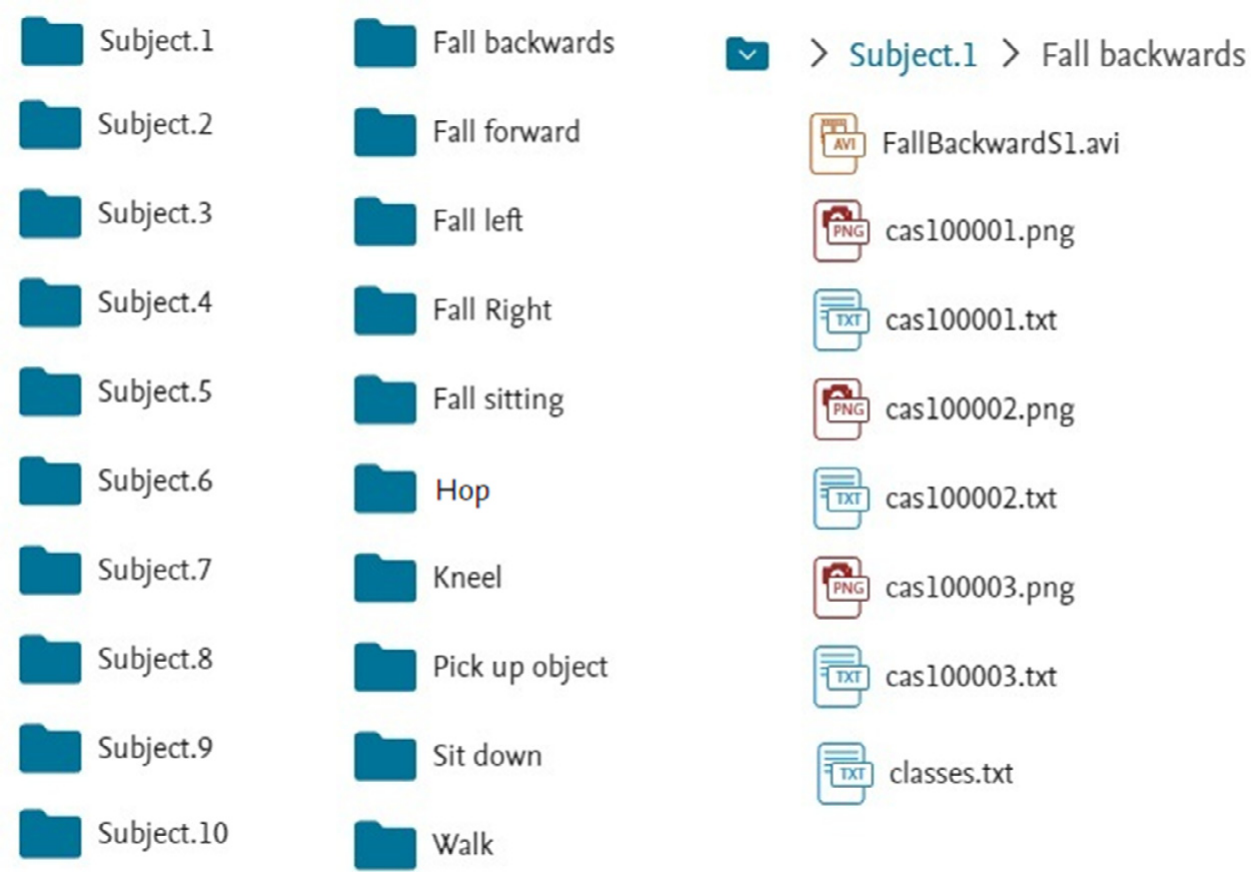
Folders for each subject and different activities of the dataset.

Fig. 5 details the folders for each subject and the different activities. An example for Subject 1, for the activity “Fall backwards”, is included. This activity has the action video in .avi format and each one of the images of the activity in .png format. Each image has the same base name, and for each image, there is a respective label. Finally, the file ``classes.txt'' specifies the name of the labels used in the images.

**Fig. 6:**
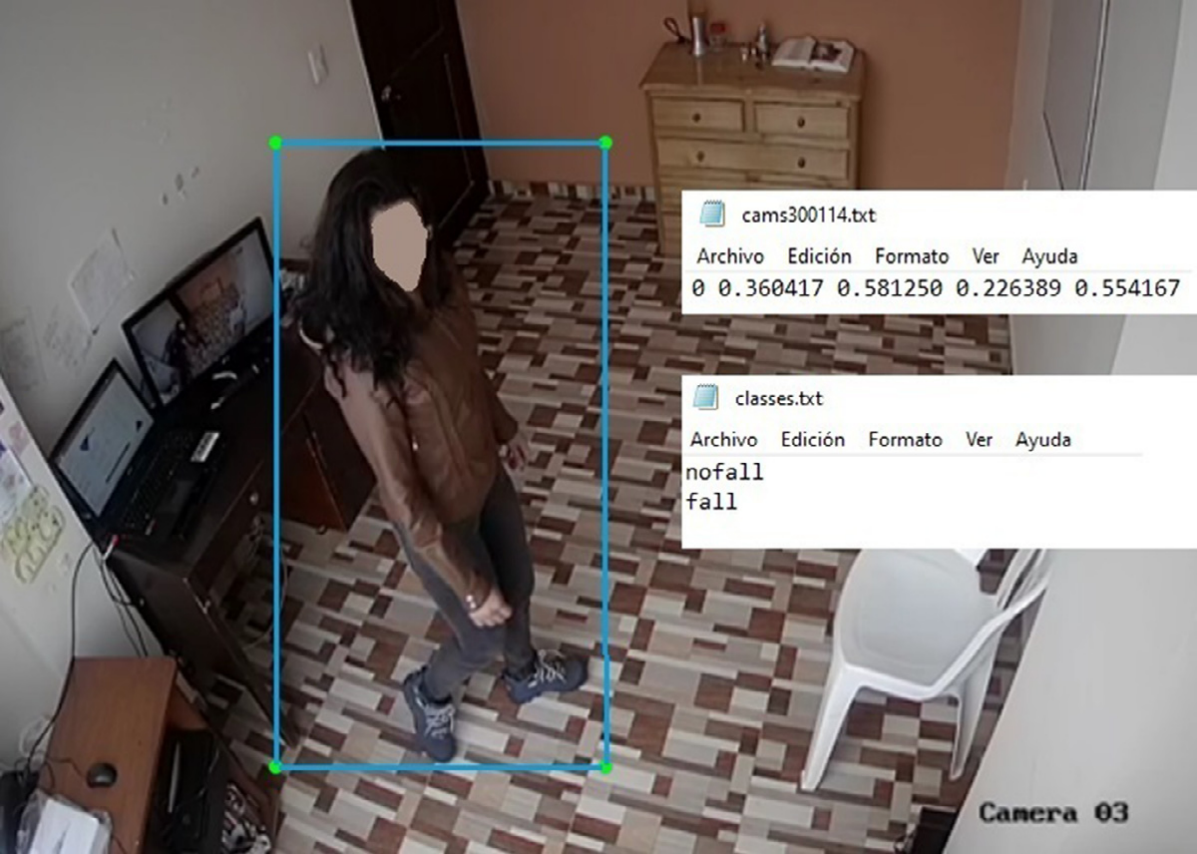
Content of the different .txt files.

The content of the different .txt files is displayed in Fig. 6. The files contain the information about the box enclosing the human silhouette and the first digit (0 or 1) identifies the label of the action being performed. The label name is defined in the file ``classes.txt'': 0 corresponds to ``nofall'' and 1 corresponds to ``fall''.

Researchers can change the tags in the ``classes.txt'' file and use the data to perform human activity recognition instead of fall recognition.

**Fig. 7:**
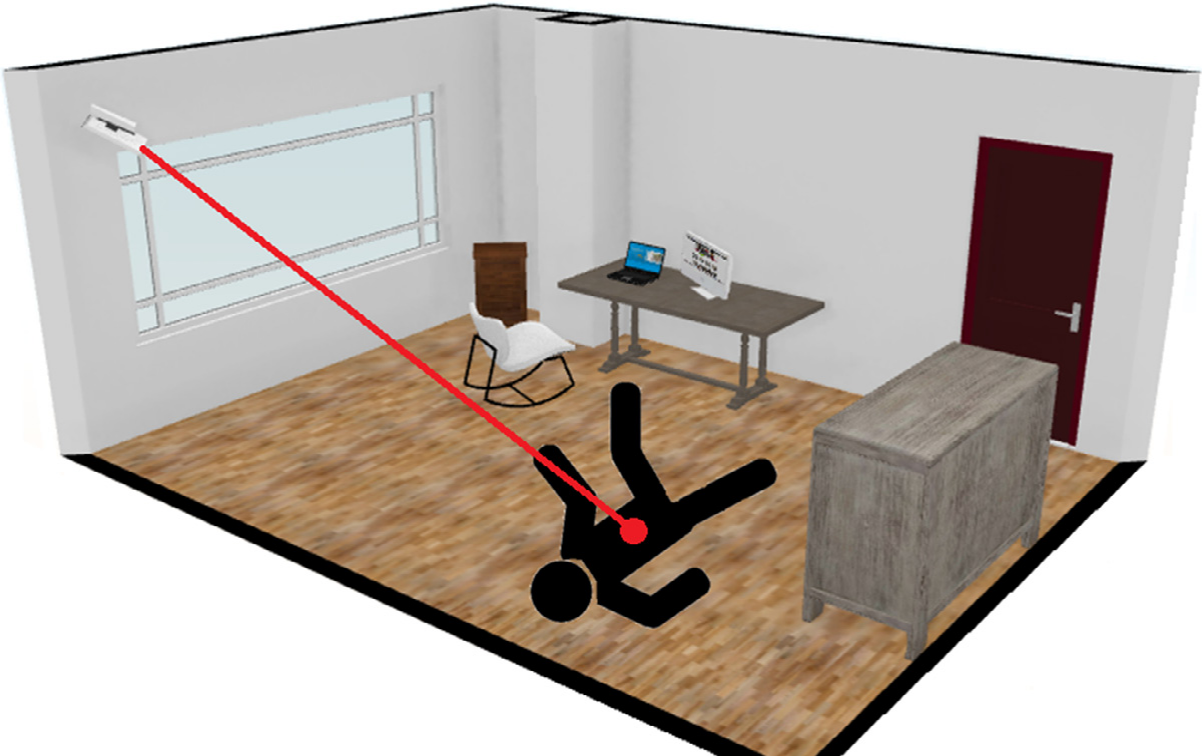
Camera-Fall distance.

**Fig. 8:**
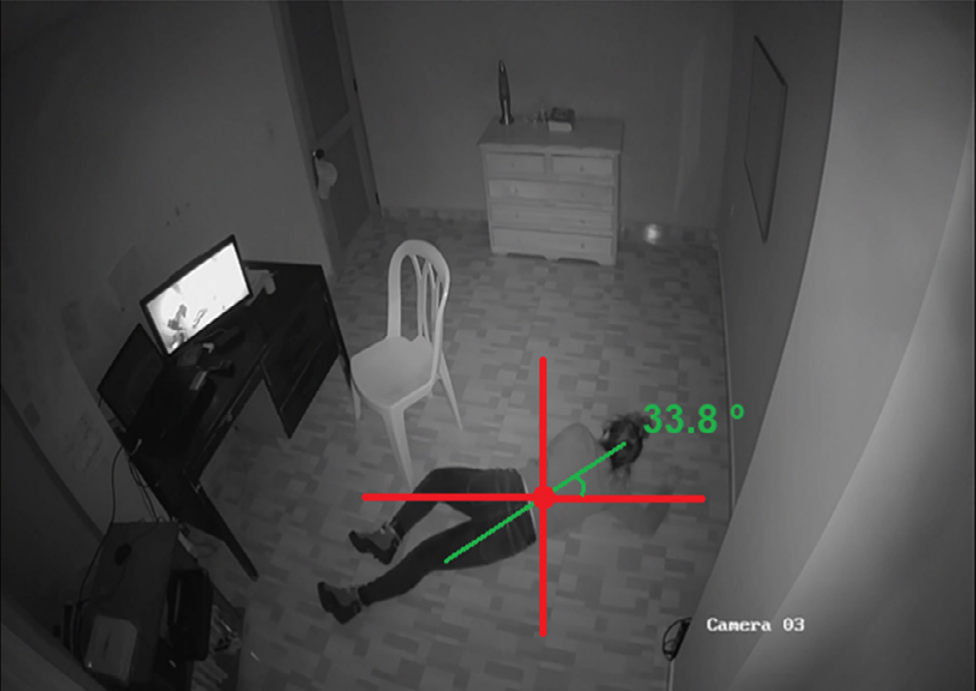
Angle of fall.

In the repository containing CAUCAFall, the file ``Dataset_details.xlsx'' is attached, which shows the actions of each subject, number of frames, distance from the camera to the centroid of the human body in each fall (see Fig. 7), angle of falls (see Fig. 8), occlusions, and the lighting conditions of the different scenarios.

## 2 Experimental Design, Materials and Methods

### 2.1 Materials

The optical system used to capture videos of human actions is composed of a HIKVISION IR camera [17], which was fixed on the upper corner of the wall in the different scenarios. This system covers large field of vision to monitor the user's activity and was connected to a HIKVISION DVR [18] with a built-in 1 TB hard disk for video storage and processing.

The DVR has continuous, manual, and motion detection modes, so recording starts when the individual enters the scene. The camera captures video at a speed of 23 fps and a resolution of 1080 × 960 pixels and supports changing illumination (i.e., natural light, low light, or no light). The IR sensor records in RGB color during natural light, while in the dark or with no light, the IR sensor provides light beams to record binary images [19].

### 2.2 Data collection protocol

1. The data collection process was performed in an uncontrolled home environment. A professional kinesiologist instructed the participants on the correct way to fall, and the most common falls in elderly people were simulated. The selected protective elements are elbow and knee pads that have strong shock absorption capacity and no restriction of movement. Each participant performed 10 activities, five ADLs, and five fall simulations. The following steps detail the phases essential to creating the dataset:
  A literature review was conducted to learn about existing datasets and their characteristics, which allowed the authors to identify their shortcomings;
2. A realistic uncontrolled environment was created, incorporating distracting elements, occlusions, environmental conditions, and changing lighting;
3. A varied population of participants in terms of age, gender, weight, height, and different dominant legs was chosen to perform the activities and the fall simulation;
4. An engineer identified the participants’ possible fall angles and determined different fall distances in front of the camera to ensure a varied dataset;
5. The data were recorded and stored for later processing.

### 2.3 Labels

In general, YOLO detectors are trained and work with labels from the COCO dataset [20] which contains 80 classes, but does not include falls. CAUCAFall includes labels so that YOLO detectors are also able to detect human falls, for which the authors manually labeled each frame of each performed activity, delimiting in each image the human silhouette, and by a visual analysis, the authors labeled the images as “fall” only when the human body is on the ground because of a fall, any other activity was labeled as ``nofall''. A total of 20,002 frames were labeled: 13,581 ADL activities were labeled ``nofall'' and 6421 were labeled ``fall'', to be able to use the algorithm for human fall recognition. Image dimensionality and correct formating were also verified, so the images contained the optimal size and dimensions to emphasize the analysis in the area of interest. This step significantly aids computer vision techniques and convolutional neural networks, reducing the computational cost.

## Ethics Statement

The data collection process was performed with the participation of human subjects, considering the Code of Ethics of the World Medical Association (Declaration of Helsinki). Each participant was notified of the research objective and the possible risks of their collaboration. In addition, the correct method to complete the activities (without putting themselves at risk) was explained to all participants. The participants were provided with protective equipment worn under their clothes. Likewise, voluntary consent forms signed by the participants were obtained.

## CRediT authorship contribution statement

**José Camilo Eraso Guerrero:** Writing – review & editing, Software, Data curation, Funding acquisition. **Elena Muñoz España:** Writing – review & editing, Conceptualization, Investigation, Supervision. **Mariela Muñoz Añasco:** Writing – review & editing, Conceptualization, Investigation, Supervision. **Jesús Emilio Pinto Lopera:** Investigation, Data curation, Methodology, Writing – review & editing.

## Declaration of Competing Interest

The authors declare that they have no known competing financial interests or personal relationships that could have appeared to influence the work reported in this paper.

## Data Availability

Dataset CAUCAFall (Original data) (Mendeley Data).
